# Early onset type 2 diabetes mellitus: an update

**DOI:** 10.1007/s12020-024-03772-w

**Published:** 2024-03-12

**Authors:** Myrsini Strati, Melpomeni Moustaki, Theodora Psaltopoulou, Andromachi Vryonidou, Stavroula A. Paschou

**Affiliations:** 1 School of Medicine, University of Patras, Patras, Greece; 2 Department of Endocrinology and Diabetes Center, Hellenic Red Cross Hospital, Athens, Greece; 3 Endocrine Unit and Diabetes Center, Department of Clinical Therapeutics, Alexandra Hospital, School of Medicine, National and Kapodistrian University of Athens, Athens, Greece

**Keywords:** Diabetes mellitus, Type 2 diabetes mellitus, Early-onset, Young

## Abstract

The incidence and prevalence of type 2 diabetes mellitus (T2DM) in young individuals (aged <40 years) have significantly increased in recent years, approximating two to threefold increase in the respective rates. Numerous risk factors including severe obesity, family history, ethnicity, maternal diabetes or gestational diabetes, and female sex contribute to a younger age of onset. In terms of pathogenesis, impaired insulin secretion is the key operating mechanism, alongside with ectopic adiposity-related insulin resistance. T2DM diagnosis in a young adult requires the exclusion of type 1 diabetes mellitus (T1DM), latent autoimmune diabetes of adults (LADA) and maturity-onset diabetes of the young (MODY). The establishment of such diagnosis is critical for prognosis, because early-onset T2DM is associated with rapid deterioration in pancreatic β-cell secretory function leading to earlier initiation of insulin therapy. Furthermore, mortality and lifetime risk of developing complications, especially microvascular, is increased in these patients compared to both later-onset T2DM and T1DM patients; also, the latter are often developed earlier in the course of disease. The management of early-onset T2DM follows the same guidelines as in later-onset T2DM; yet patients aged 18–39 years are underrepresented in the big clinical trials on which the development of guidelines is based. Finally, young people with T2DM face significant challenges associated with social determinants, which compromise their adherence to therapy and induce diabetes distress. Future research focusing on the pathogenesis of β-cell decline and complications, as well as on specific treatment shall lead to better understanding and management of early-onset T2DM.

## Introduction

Diabetes mellitus (DM) is a global pandemic, currently affecting 1 in 10 individuals aged 20–79 years and ranking among the leading causes of premature mortality. Its global incidence and prevalence are continuously increasing, with the latter being anticipated to reach 784 million by 2045 [1]. Type 2 diabetes mellitus (T2DM) is characterized by insulin resistance and insufficient insulin secretion [2] and accounts for more than 90% of all types of DM worldwide [1]. Although previously known as a metabolic disorder occurring in middle and late adulthood, the increasing rates of T2DM in children, adolescents, and young adults in recent years and especially after the 2000s, as reflected by almost tripling of the respective standardized incidence ratio in individuals up to the age of 40 years, raises serious public health concerns [1–7]. Accumulating evidence indicates that early-onset T2DM is associated with a more severe disease phenotype, characterized by faster decline in β-cell secretory function, leading to necessitation of insulin therapy earlier in the course of disease, alongside with increased lifetime risk of developing unfavorable long-term outcomes [2]. Despite the increased prevalence and severity of this clinical entity, several pathogenetic and clinical aspects are not entirely understood. This review aims to explore the risk factors, pathophysiology, clinical presentation, differential diagnosis, complications, and management of early-onset T2DM, based on evidence acquired from clinical studies over the last 2 decades.

## Risk factors

The upward trend of early-onset T2DM is associated with numerous different risk factors, which demonstrate the disease’s multifactorial character. Worryingly, obesity, showing escalating rates especially among young populations and being mainly driven by unhealthy lifestyle habits, appears to be the major contributor [2]. In a study aiming to assess the phenotypic characteristics and risk factors leading to early-onset T2DM (<40 years of age) conducted by Lascar et al. 95% of the participants were found to be either overweight or obese [8]. Similarly, in a nationwide-based study in Israel of ~1.5 million adolescents, Twig et al. described that severe obesity (body mass index, BMI > 35 kg/m^2^) increases the risk for T2DM in early adulthood in both sexes [9].

Furthermore, genetic predisposition and family history of T2DM seem to be strong predictors of early presentation of the disease, with 60% of patients having one parent affected by T2DM [10]. Unlike type 1 diabetes mellitus (T1DM), where ∼90% of patients have a negative family history [11], 74–100% of children presenting with T2DM are estimated to have a 1st or 2nd-degree relative with T2DM [12].

Notably, early-onset T2DM is highly represented in people originating from specific ethnic groups, such as Indigenous Australians, Pima Indians, Native Americans, and Canada’s First Nation people, a fact that might be linked either to genetic predisposition or to lower socioeconomic status [10]. Τhe Progress in Diabetes Genetics in Youth (ProDiGY) Consortium collected data from three studies (TODAY Study, SEARCH Study, and T2D-GENES) in order to explore the effect of various genetic variants predisposing specifically to youth-onset T2DM and discovered seven genome wide associations. [13].

Moreover, Pettitt et al. in the SEARCH for diabetes study found that early life determinants affecting the intrauterine environment, such as maternal gestational DM and obesity, are associated with increased risk of developing T2DM earlier in life [14]. In addition, a systematic review and meta-analysis demonstrated that newborns small for gestational age (SGA), compared to the ones with adequate size for gestational age, have an increased risk (2.33-fold higher) of developing T2DM in childhood or adolescence years [15].

Finally, female sex, especially if polycystic ovarian syndrome (PCOS) is present, predisposes to earlier presentation of T2DM [8]. Last but not least, other, well recognized risk factors for late-onset T2DM such as non-alcoholic fatty liver disease (NAFLD), hypertension [16], dyslipidemia, and albuminuria are frequently encountered among young patients with T2DM as well (Table 1) [10].

## Pathophysiology

Compared to T1DM in the same age and T2DM in middle and late adulthood, early-onset T2DM, including both children and young adults, is shown to have higher rate of complications and more rapid deterioration of β-cell function respectively [1, 10, 17]. Although the pathophysiology and precise pathways through which T2DM develops in young people are not fully described yet, the overall etiology is similar to that of later-onset T2DM, including insulin resistance and β-cell dysfunction [10].

### Insulin resistance

Insulin resistance is a metabolic state characterized by great complexity, induced by multiple suggested pathways.

In the first place, obesity is more common in early-onset T2DM compared to later-onset T2DM (95% vs. 50%) and it is considered to be one of the key drivers in developing the early-onset phenotype of the disease [10]. Indeed, BMI and age of T2DM onset are shown to have a strong inverse correlation [18].

More important than obesity per se seems to be the unfavorable distribution of adipose tissue, characterized by decreased subcutaneous adipose tissue and increased intramyocellular and intrahepatic lipid content, which is also more prominent in patients with early-onset T2DM. In fact, intrahepatic fat, which is emerging as the most important marker of insulin resistance, is threefold higher compared to later-onset T2DM and BMI-matched peers without diabetes [2, 19]. The ectopic, intracellular accumulation of excess lipid metabolites in skeletal muscle and liver leads to post-receptor impairment of insulin signaling. In skeletal muscle, there is inhibition of insulin-receptor substrate tyrosine phosphorylation and hence of the following phosphatidylinositol 3 kinase activity and glucose transporter 4 transportation to the cell surface, resulting into decreased glucose uptake and glycogenesis, which is actually considered as the primary metabolic defect in T2DM pathogenesis [20]. Similarly, in liver, there is inhibition of glycogen synthase activity and stimulation of gluconeogenic enzymes expression, leading to attenuated glycogenesis and enhanced gluconeogenesis [21].

Furthermore, youth populations with T2DM show evidence of systemic inflammation, which appears to be interrelated with hepatokines’ and adipokines’ profile. According to two cross-sectional studies comparing obese adolescents with and without T2DM matched for BMI, sex, and age, the former demonstrate higher levels of the hepatokines fibroblast growth factor 21 (FGF21) and fetuin-A, as well as of high-sensitivity C-reactive protein (hsCRP), tumor necrosis factor alpha (TNF-α) and interleukin 1 beta [22, 23]. Interestingly, there is a positive correlation between FGF21 and hsCRP and a negative correlation between leptin and TNF-α [23]. Given that FGF21 is known to have an insulin-sensitizing effect, these findings suggest an inflammation-induced FGF-resistant state, in the absence of leptin resistance in this setting [23]. In turn, fetuin-A is associated with intrahepatic fat and suggested to induce insulin resistance in parallel with promoting low-grade inflammation, leading to a mutually amplifying loop between them [22].

Additionally, intrauterine environment emerges as a key player of insulin resistance in this setting. According to the Hyperglycemia and Adverse Pregnancy Outcome Follow-up Study (HAPO FUS), there is linear association between maternal and child glycemia across the spectrum of glucose levels, alongside with inverse association between maternal glucose levels at all time points of 2-h oral glucose tolerance test and child insulin sensitivity, assessed by Matsuda index, independently of mother or child BMI and family history. These results highlight the contribution of exposure to higher levels of glucose in utero to development of insulin resistance earlier in life [24]. Moreover, low birth weight (LBW) and SGA were also associated with 0.20 increased mean levels of homeostasis model assessment of insulin resistance in a systematic review and meta-analysis. The exact mechanisms underlying this phenomenon remain unexplained, but several hypotheses have been developed including overfeeding tendency during perinatal period of LBW newborns (“the early catch-up theory”) and metabolic stress events in utero causing aberrant vasculature and endocrine dysregulation (“the fetal programming theory”). [15].

Moreover, hormonal changes throughout puberty are also considered to amplify insulin resistance. The expected surge in growth hormone (GH) followed by extensive lipid breakdown and increase in free fatty acids concentration in the bloodstream comprises the main mechanism of short-term increase in insulin resistance during this period [25].

Finally, several studies describe a positive association between metabolic disease and sleep deprivation, with the latter being prominent among adolescents in recent years. Chronic sleep deprivation is associated with higher cortisol levels, inflammatory markers, and decreased testosterone levels. Additionally, by altering the expression of several appetite-related hormones (e.g., orexin, ghrelin) sleep deprivation is described to cause an imbalance in central appetite regulation controlled by the hypothalamus, and therefore to increase the risk of obesity [26].

### β-cell dysfunction

In order to compensate for the increased insulin resistance, β-cells initially increase insulin secretion. However, over time, the number and secretory response of healthy β-cells decreases due to glucolipotoxicity, endoplasmic reticulum stress, mitochondrial dysfunction and inflammation, which crosstalk with genetic and epigenetic factors [27]. The substantial difference between early- and later-onset T2DM is that the impairment of β-cell function progresses more rapidly, highlighting that impaired insulin secretion is the key operating pathophysiologic mechanism. In particular, the yearly β-cell function deterioration in early-onset T2DM was reported to be 20–35% by The Multi-Ethnic Treatment Options for T2DM in Adolescents and Youth (TODAY) study of 699 youth and adolescents with T2DM (mean age 14 years, USA), much higher compared to 7% per year in later-onset T2DM [28].

The exact mechanisms underpinning the accelerated loss of β-cell function in early-onset T2DM are not entirely understood. A widely accepted hypothesis in explanation of this phenomenon is that insulin hypersecretion, assessed by insulin and C-peptide levels, at the initial stages of impaired glucose tolerance (IGT) or newly diagnosed T2DM is more pronounced in children and adolescents compared to older adults, and thus, might lead to faster β-cell exhaustion [29]. In this regard, increases in hormones related to growth and puberty, such as GH and sex steroids have been shown to overstimulate β-cells, which express GH, estrogen, and androgen receptors [25, 30]. Considering that insulin sensitivity decreases by 30–50% during puberty even in healthy, normal weight children, the above hormonal stimulation most likely comprises a compensatory mechanism, which, however, may not be adequate in individuals prone to β-cell dysfunction, who may present with insulin deficiency during this period [27].

Additionally, intrauterine environment and birth weight appear to affect substantially β-cell function besides insulin sensitivity. According to HAPO FUS, maternal glycemia is inversely associated with child’s disposition index (DI), a measure of pancreatic β-cell function which is shown to predict the progression to T2DM [24]. Similarly, obese children born SGA demonstrate deficit in early insulin response and DI [31]. Apart from stressing β-cell via increasing insulin resistance, pathological intrauterine environment appears to affect β-cell function per se. Data from a large study comparing 568 non-diabetic offspring of mothers and fathers with early-onset T2DM, for the same degree of insulin resistance, the former had lower early insulin response, highlighting the importance of intrauterine hyperglycemia in fetal β-cell programming even among patients with equal genetic predisposition to early-onset T2DM [32]. Similarly, a previous small study dated in 1970s, which compared pancreatic biopsies of SGA and normal weight babies of similar gestational age that died within the first 48 h after birth, indicated that the former had decreased fetal endocrine pancreatic tissue and percentage of β-cells [33].

Furthermore, impaired proinsulin processing, reflected at increased proinsulin levels and proinsulin-to-insulin ratio, has been demonstrated in two studies in young subjects with T2DM, including the large-scale TODAY study [34]. According to the latter, impaired proinsulin processing comprises an early predictor of glycemic control deterioration [35]. The underlying mechanisms have not been elucidated. However, considering that hyperproinsulinemia does not precede the onset of T2DM points away from the possibility of being compensatory in young patients [34].

Genetically-wise, there are few data regarding specific genotypes linked to early-onset T2DM. Particularly, rs3738435 variant of muscarinic acetylcholine receptor subtype 3 (CHRM3) gene is associated with decreased acute insulin response and increased risk of early-onset T2DM in Pima Indians [36], and rs7903146 variant of transcription factor 7-like protein 2 (TCF7L2) is linked to decreased β-cell responsivity and DI, impaired proinsulin processing, and increased risk of IGT and T2DM in obese adolescents of Caucasian and African American origin [37]. Regarding the contribution of epigenetic factors, rodent data indicate that depletion of m6 mRNA methylation leads to islet phenotype of early-onset T2DM, via inducing cell-cycle arrest and impairing insulin secretion via inhibition of protein kinase B (AKT) phosphorylation and pancreatic duodenal homeobox 1 (PDX1) protein levels [38].

In terms of environmental factors, as recently reviewed, adequate intake of specific micronutrients, such as vitamin D, calcium, vitamin A, zinc, and iron is important for the preservation of β-cell function. Interestingly, some of these factors, such as vitamins A and D appear to be essential for the development of fetal pancreatic islets. Nevertheless, the role of nutrients in pathogenesis of early-onset T2DM is scarcely studied [27]. Finally, recently published data reveal that the second phase of insulin secretion is inversely correlated with bedtime, which is later in patients with early-onset T2DM than in those with later-onset T2DM [39].

### Compared to T1DM

Early-onset T2DM and T1DM are both a result of the loss of function and mass of β-cells in pancreatic islets, but their underlying pathogenic mechanisms are distinguished by fundamental differences. In early-onset T2DM, β-cell failure is the end pathophysiological stage, preceded by insulin hypersecretion which initially compensates for the increased insulin resistance mainly on the grounds of obesity, ectopic adiposity, puberty, and inflammation. The faster exhaustion of β-cell reserve in this setting is multifactorial, being related to higher insulin resistance, fetal programming in utero, genetic, epigenetic, and environmental parameters [27]. On the contrary, in T1DM, β-cell failure due to autoimmune-mediated β-cell apoptosis is the initial event in pathogenesis. More specifically, the key mechanism underlying T1DM is driven by the presence of autoreactive T-lymphocytes and macrophages which target β-cell surface antigens and release cytokines in the microenvironment of the pancreatic islets resulting in an inflammatory reaction, called “insulitis”, and progressive β-cell death. In most cases, these events cause a total deficiency of insulin secretion and therefore lifelong exogenous insulin replacement therapy is required [40, 41]. Despite the increased evidence of insulin resistance in young patients with T1DM leading to overlap of clinical phenotype between the two types of DM, this event is not intrinsic to pathophysiology; rather, it is secondary to obesity, inflammation, exogenous insulin treatment in parallel with decreased insulin delivery to portal circulation, and, to certain degree, determined by genetics and ethnicity [42].

## Clinical presentation and differential diagnosis

Early-onset T2DM is strongly associated with obesity, metabolic syndrome features, insulin resistance, family history, and necessitates faster progression to insulin therapy, i.e., in 2–5 years after diagnosis in >50% of patients [2]. Recently, a study led by Baek et al. demonstrated that patients with early-onset T2DM present with higher levels of fasting glucose and HbA1 at the time of diagnosis and have poorer glycemic control and higher glycemic variability compared to middle- and older- onset age groups [43].

The classification of different forms of diabetes in young populations presents significant challenges, as the differential diagnosis spectrum is wider than in older populations. Early-onset T2DM overlaps with clinical patterns commonly seen both in T1DM, latent autoimmune diabetes of adults (LADA), maturity-onset diabetes of the young (MODY). For instance, the more aggressive phenotype of the disease, accompanied by early insulinopenia and initiation of insulin therapy, overlaps with clinical characteristics of T1DM, LADA, and MODY types 1 and 3.

Unlike T2DM, immune-mediated diabetes (T1DM, LADA) is identified by the presence of autoimmune markers. The presence of two or more autoantibodies that target antigens of the β-cell secretory granules is indicative of either T1DM or LADA. These include islet cell autoantibodies, glutamic acid decarboxylase autoantibodies, insulin autoantibodies, tyrosine phosphatases islet antigen 2 autoantibodies (IA-2A and IA-2β), and also zinc transporter 8 autoantibodies [44]. Although β-cell destruction and corresponding insulin reserve vary among individuals with autoimmune diabetes, by the time that full-blown picture of insulinopenia is established, plasma C-peptide level is usually undetectable and may be used to differentiate immune-mediated diabetes from other DM types [44, 45], except from the honeymoon phase of LADA. Importantly, considering the continuously increasing prevalence of obesity in the general population, BMI is not considered a reliable feature to differentiate between T2DM from T1DM and should not preclude autoimmune diabetes testing [46]. Additionally, due to overlapping genetic susceptibility, autoimmune diabetes is often associated and can co-exist with additional autoimmune disorders such as autoimmune thyroid disease, celiac disease, autoimmune hepatitis, and others; thus, should be suspected in patients with an autoimmune background [47] .

**Table 1:** Modifiable and non-modifiable risk factors associated with early-onset T2DM

Modifiable	Non-modifiable
▪ Overweight & obesity ▪ Lifestyle habits ▪ Socioeconomic disadvantage ▪ Non-alcoholic fatty liver disease (NAFLD) ▪ Hypertension ▪ Dyslipidemia ▪ Albuminuria	▪ Strong family history of T2DM ▪ Genetics ▪ Specific ethnic groups ▪ Early life determinants ▪ Female sex ▪ Polycystic ovary syndrome (PCOS)

**Table 2:** I. Studies investigating the association between age at T2DM onset, complications, and mortality and II. Studies comparing the prevalence of complications between early-onset T2DM and later-onset T2DM or T1DM in similar ages

Study/year published	*N*	Subject(s)	Type of the study	Length of follow-up	Results
**I. Studies investigating the association between age at T2DM onset, microvascular, macrovascular complications, and mortality**					
Impact of age at diagnosis and duration of type 2 diabetes on mortality in Australia/2018 [38]	743,709	Patients with T2DM, registered between 1997 and 2011 on National Diabetes Service Scheme in Australia, mean age 60.2 years, mean age at diagnosis 58.6 years	Cohort, retrospective, longitudinal, comparative study	7.2 years	Younger age of onset associated with ↑ mortality: 10 years earlier diagnosis linked to 1.2–1.3 times ↑ in all-cause mortality and 1.6 times ↑ CVD mortality
Age at diagnosis of type 2 diabetes mellitus and associations with cardiovascular and mortality risks/2019 [37]	214,278	Patients with T2DM without CVD, from Swedish National Diabetes Registry, ages 9–109 years, mean age at diagnosis 61.79 years	Cohort, retrospective, longitudinal study with matched healthy controls	5.63 years	Patients diagnosed at ≤40 years ↑ excess risk compared to controls for total mortality, CV mortality, non-CV mortality and other CVD outcomes
Impact of age at type 2 diabetes mellitus diagnosis on mortality and vascular complications/2021 [36]	1,325,493	Patients with T2DM from 30 countries, mean age from 21.6 to 67.4 years	SR/MA of 26 observational studies	n/a	1-year ↑ at age of diagnosis associated with 4%, 3%, 5% ↑ in all-cause mortality, macrovascular disease, microvascular disease, respectively
TODAY Study Group: long-term complications in youth-onset type 2 diabetes/2021 [34]	500	Patients with T2DM mean age 26.4 ± 2.8 years, mean time since diagnosis 13.3 ± 1.8 years	Cohort, prospective, longitudinal, observational	10.2 years	Rapid accumulation: 60.1% of participants developed ≥1 and 28.4% ≥ 2 complications
**II. Studies comparing the prevalence of complications between early-onset T2DM and later-onset T2DM or T1DM in similar ages**					
Prevalence of diabetes complications in adolescents with type 2 compared with type 1 diabetes/2006 [48]	1433	Patients with T1DM (<18 years) mean age at diagnosis 8.1 years and median duration 6.8 years	Cohort, retrospective, longitudinal, comparative study	n/a	↑ Rates of microalbuminuria and hypertension in T2DM vs. T1DM subject (28 and 36% vs. 6 and 16% respectively) and ↑ rates of retinopathy in T1DM vs. T2DM subject (20 vs. 4%, *P* = 0.04), ↔ neuropathy rates
	68	Patients with T2DM (<18 years) mean age at diagnosis 13.2 years and median duration 1.3 years			
Long-term complications and mortality in young-onset diabetes/2013 [49]	470	Patients with T1DM median duration 4.0 years	Cohort, retrospective, longitudinal, comparative study	23.4 for T1DM	Twofold increase in case fatality in T2DM vs. T1DM subject, ↑ prevalence of albuminuria, neuropathy, and macrovascular disease in T2DM vs. T1DM subject, ↔ nephropathy, ↔ retinopathy
	354	Patients with T2DM median duration 3.9 years		21.4 years for T2DM	
		diagnosed between 15–30 years (both)			
Complication characteristics between young-onset type 2 vs. type 1 diabetes in a UK population/2015 [50]	760	Patients with T1DM mean age at diagnosis 25.8 ± 6.9 and median duration 20.4 ± 12.9 years	Cohort, retrospective, cross-sectional, comparative study	n/a	↑ Risk of CVD and neuropathy in T2DM vs. T1DM subject in all diabetes durations, ↔ retinopathy
	527	Patients with T2DM mean age at diagnosis 32.5 ± 5.9 and median duration 15.0 ± 10.3 years			
An inverse relationship between age of type 2 diabetes onset and complication risk and mortality: the impact of youth-onset type 2 diabetes/2016 [43]	354	Patients with T2DM diagnosed between 15 and 30 years, mean age at diagnosis 25.6 years	Cohort, retrospective, comparative study	n/a	↑ Risk of renal complications and neuropathy in T2DM_15–30_ vs. T2DM_40–50_ subject, ↔ retinopathy
	1062	Patients with T2DM diagnosed between 40 and 50 years, mean age at diagnosis 45.2 years (matched for duration, glycemic control, and male: female ratio)			Inverse relationship between age of onset and SMRs^a^ with the highest in T2DM_15–30_ (3.4 [95% CI 2.7–4.2])
Association of type 1 diabetes vs. type 2 diabetes diagnosed during childhood and adolescence with complications during teenage years and young adulthood: the SEARCH for Diabetes in Youth Study /2017 [46]	1746 272	Patients with T1DM Patients with T2DM diagnosed <20 years with median duration 7.9 years (both)	Cohort, prospective, longitudinal, comparative study	2002–2015	↑ Prevalence of complications in T2DM vs. T1DM subject: CKD (19.9% vs. 5.8%), retinopathy (9.1% vs. 5.6%), peripheral neuropathy (17.7% vs. 8.5%) after adjustment for risk factors, ↔ arterial stiffness, ↔ hypertension
Younger-onset vs. older-onset type 2 diabetes: clinical profile and complications/2017 [44]	267	Patients with T2DM diagnosed ≤25 years, mean age at diagnosis 21.3 ± 3.6 years	Cohort, cross-sectional comparative study	n/a	Prevalence of retinopathy ↑ in T2DM_≤25_ vs. T2DM_≥50_ subject (47.6 vs. 31.0%), prevalence of neuropathy and peripheral vascular disease ↑ in T2DM_≥50_ vs. T2DM_≤25_ subject (41.8 vs. 9.2% and 6.2 vs. 1.2%, respectively)
	267	Patients with T2DM diagnosed ≥ 50 years, mean age at diagnosis 57.2 ± 5.7 years (matched for duration)			
Longitudinal changes in arterial stiffness and heart rate variability in youth-onset type 1 vs. type 2 diabetes: the SEARCH for Diabetes in Youth Study/2022 [47]	949 210	Patients with T1DM Patients with T2DM both diagnosed <20 years with median duration 7.9 years	Cohort, prospective, longitudinal comparative study	4.6 ± 1.1 years	↑ Arterial stiffness (pulse wave velocity, augmentation index) and HRV abnormalities over time in T2DM vs. T1DM subject (especially if metabolic syndrome signs present)
Prevalence and risk of diabetic complications in young-onset vs. late-onset type 2 diabetes mellitus/2022 [42]	1791	Patients with T2DM diagnosed <40 years, mean age at diagnosis 33.8 years	Cohort, retrospective, longitudinal, comparative study	n/a	↑ Prevalence of complications in T2DM_<40_ vs. T2DM_≥40_ subject attributable to longer duration of disease, except prevalence neuropathy: remained significantly ↑ after adjustment for duration
	8656	Patients with T2DM diagnosed ≥40 years, mean age at diagnosis 53.3 years			

The table is divided in two subsections, the first presenting studies which have investigated the association between age at T2DM diagnosis and microvascular, macrovascular complications and mortality, and the second presenting studies which have compared the prevalence of complications between early-onset T2DM and either later-onset T2DM or T1DM in similar ages. Studies are presented in chronological order in each subsection. The data for each study are classified according to number of participants, study population(s), type of the study, length of the follow-up and relevant results

*CVD* cardiovascular disease, *CV* cardiovascular, *SR/MA* systematic review and meta-analysis, *CKD* diabetic kidney disease, *HRV* heart rate variability, *n/a* non available, ↑: significant increase, ↓: significant decrease, ↔: no change

^a^Standardized mortality ratios [34, 36–38, 42–44, 46–50]

MODY, the most common type of monogenic diabetes, is caused by a single-gene mutation resulting in impaired β-cell function and decreased insulin secretion. Similarly to immune-mediated diabetes and early-onset T2DM, MODY also typically presents during adolescence or early adulthood years. At least 14 subtypes have been identified to date, based on the specific mutated gene with the majority of cases being classified into three main subtypes (MODY 1–3). Clinical features, age of onset, and management differ depending on specific gene affected. Notably, MODY 1 and MODY 3 present with phenotype of insulin deficiency, including polyuria, polydipsia, and weight loss. Their substantial difference with both early-onset T2DM and T1DM is that insulin secretion is restored by treatment with sulfonylureas. In addition, patients with MODY 1 usually have history of macrosomia and neonatal hyperglycemia, which is also common in patients with early-onset T2DM exposed to hyperglycemia in utero; however, in MODY 1 cases such manifestations may occur even in the absence of maternal or gestational DM. Contrary to the above subtypes, MODY 2 presents with mild fasting hyperglycemia, neither requiring treatment nor being associated with chronic complications; therefore, it can be easily distinguished from early-onset T2DM based one the absence of progression. Importantly, the autosomal dominant transmission in most forms of MODY overlaps with the strong association between early-onset T2DM and positive family history. In addition, similarly to immune-mediated diabetes, overweight or obesity may also be present in patients with MODY and should not preclude genetic testing for MODY panel, especially if no other markers of metabolic syndrome are present, not all affected relatives are obese and autosomal dominant mode of inheritance is present [44, 48]. Considering these overlapping features as well as the possibility of inadequate genetic testing, MODY patients, although representing a small fraction of all diabetes types, may remain underrecognized. Indeed, 50–90% of MODY cases are reported to be misdiagnosed as T1DM or T2DM [49].

## Complications and mortality

Given the rising incidence and the faster progression of T2DM in young populations, several studies during the past few years aimed to investigate the association between the early age at diagnosis and the risk of developing several complications, compared to later-onset T2DM or T1DM. Several questions have also been stated about whether the possible higher risk of morbidity and mortality is attributed to a more aggressive phenotype of the disease, a more prolonged duration of diabetes, or both. The contribution of various risk factors to the development of some complications was also examined in several studies (Table 2).

In the prospective, follow-up study conducted after the completion of TODAY study, aiming to assess the occurrence of T2DM-related complications among youth (mean age 26.4 ± 2.8 and mean time since diagnosis 13.3 years), at least one microvascular complication developed in 60.1% and at least two in 28.4% of the participants. Complications examined included hypertension, dyslipidemia, and microvascular complications such as neuropathy, nephropathy, and retinopathy [50]. Moreover, according to the SEARCH for Diabetes in Youth Study hyperglycemia was positively associated with dyslipidemia in adolescents with T2DM; in fact, there was a 20% and 13% higher risk of either deterioration or maintaining abnormal LDL-C and triglycerides levels respectively for each 1% increase in glycated hemoglobin (HbA1c) in young adolescents with T2DM [51]. However, this correlation does not apply exclusively in early-onset T2DM populations. In a study including 1747 T2DM patients of all age ranges (average 58.6 years), a positive association between blood glucose levels and TG, LDL-C, TG/HDL-C, and LDL/HDL-C was also established [52]. Additionally, in a meta-analysis integrating data from 26 observational studies comprising, in total, over a million of participants worldwide, Nanayakkara et al. established an inverse association between age at T2DM diagnosis and all-cause mortality, macrovascular, and microvascular disease [53].

Furthermore, a study by Sattar et al. collecting data from the Swedish National Diabetes Registry from 1998 to 2013 aimed to examine the association between age at T2DM diagnosis and risk of cardiovascular disease (CVD) and mortality and reported higher risk of all analyzed outcomes in patients with younger age at T2DM diagnosis [54]. Similarly, in a population-based cohort in Australia by Huo et al. younger age of onset is associated with all-cause mortality as well as with higher rates of cardiovascular (CV) mortality, emphasizing the importance of effective CVD risk factor management [55].

Apart from the traditional complications of diabetes, additional complications such as subclinical hearing impairment and infertility, are also described in young patients with T2DM [2, 56, 57]. Such complications might remain underrecognized but can substantially affect the quality of life of these patients. In addition, early-onset T2DM patients may be more prone to mental health disorders. In a study of 1114 patients, Riaz et. al observed that the diagnosis of T2DM in patients <40 years of age is associated with an increased risk of developing depression [58].

### Compared to later-onset T2DM

In an observational study of 10,447 patients with T2DM, Cho et al. aimed to compare the prevalence of diabetic retinopathy, neuropathy, nephropathy, and carotid artery plaque formation between patients with early (diagnosed <40 years) and late (diagnosed >40 years)-onset T2DM and found higher prevalence of these complications in the early-onset group. However, after adjustment for the duration of the disease, only the prevalence of neuropathy remained significantly higher [59].

To minimize the effect of disease duration, Al-Saeed et al. compared the prevalence of complications and mortality in patients with early-onset T2DM (diagnosed between 15 and 30 years) to patients with later-onset T2DM (diagnosed between 40 and 50 years), matched for disease duration, glycemic control, and male to female ratio. As demonstrated, albuminuria and neuropathy were more prominent in the young-onset group, without differences in retinopathy among the two groups. This outcome is attributed to the “inherent” morbidity of having T2DM at a younger age by the authors. Interestingly though, the younger-onset patients were less frequently treated for hypertension and dyslipidemia in this study, possibly reflecting another possible cause. In addition, the standardized mortality ratio (SMR) was shown to be markedly higher in the young-onset group, reaching sixfold increase compared to that of the background population by the age of 40 years. This observation indicates that morbidity and mortality are not only higher in the young-onset group, but also occur early [60].

Furthermore, Unnikrishnan et al. also compared the risk factors and complications between T2DM patients diagnosed ≤25 years and ≥50 years and described poorer glycemic and lipid control in the younger-aged group. After adjusting for various risk factors, the early-onset group was shown to have higher risk of developing retinopathy [61]. Finally, according to a retrospective study in China led by Huang et al. early age of T2DM onset increases the risk of developing long-term microvascular, but not macrovascular complications [62].

### Compared to T1DM

In the observational SEARCH for Diabetes in Youth Study, conducted from 2002 to 2015 and comprising 2018 participants with type 1 and 2 diabetes with age of diagnosis <20 years, Dabelea et al. demonstrated that the prevalence of diabetic kidney disease (CKD), retinopathy and peripheral neuropathy was higher among the early-onset type 2 group, after adjustment for differences in various risk factors. No significant differences in arterial stiffness and hypertension were reported compared to the T1DM group [63].

In a more recent longitudinal study conducted also in the context of the SEARCH Study group, Shah et al. compared the arterial stiffness and heart rate variability (HRV) in participants with young-onset type 1 vs. type 2 diabetes and demonstrated that both factors worsened moreover time in patients with T2DM, and especially in those with signs of metabolic syndrome [64].

Eppens et al. compared the prevalence of various complications among patients with T1DM and T2DM diagnosed before 18 years and reported higher rates of microalbuminuria and hypertension in T2DM patients, higher rates of retinopathy in T1DM patients and no differences in neuropathy rates. The only risk factor reported to have an association with higher albuminuria rates in the T2DM group was higher HbA1c. [65].

Constantino et al. also aimed to compare long-term outcomes and survival rates between patients with T1DM and T2DM with age of onset ranging from 15 to 30 years. Similarly, twofold higher mortality rates were demonstrated in T2DM patients compared to T1DM peers, with similar age and duration. A significantly higher prevalence of albuminuria as well as macrovascular risk factors and complications were described in the T2DM cohort, but no differences in retinopathy and renal disease [66].

In a cross-sectional study in the UK, Song et al. also compared the risk of complications between the groups of young-onset T2DM and T1DM. After adjusting for various risk factors, the T2DM group was shown to be more predisposed to cardiovascular disease and neuropathy, and to have similar risk for developing retinopathy compared to the T1DM group [67].

## Management

Little evidence exists to specify the therapeutic management for early-onset T2DM. Thus, treatment planning is mainly based on existing T2DM evidence-based protocols [2]. Importantly, a recent study by Sargeant et al. reported that adult patients with early-onset T2DM (aged 18–39 years) are severely underrepresented in prominent clinical trials on which diabetes management guidelines are based [68]. A multidisciplinary care team, including pediatric and adult endocrinologists, educators, dietitians, psychologists, and other specialists is essential in order to achieve the best possible outcomes [69].

With the aim of decreasing the risk of the severe and rapidly developing complications of the disease, achieving adequate glycemic control is fundamental [2]. Lifestyle interventions, including achieving/ maintaining optimal weight, healthy dietary habits, regular exercise, smoking cessation, and limiting alcohol consumption are key pillars of treatment and together with diabetes self-management education should be prioritized and considered as the first line and essential component of the patient’s management plan [70].

Whether remission of early-onset T2DM is feasible if proper management of lifestyle risk factors is achieved is a matter of great importance. The Diabetes Remission Clinical Trial (DiRECT study) aimed to examine whether an intensive weight management program (total diet replacement) delivered in routine care, along with discontinuation of all diabetes medication, could attain remission of T2DM, defined as HbA_1c_ < 6.5%. Participants included in this study aged 20–65 years, were diagnosed within 6 years, had BMI of 27–45 kg/m^2^ and were not treated with insulin. Primary results at 12 months were weight loss of ≥15 kg in 24% and T2DM remission in 46% of participants in the intervention group (vs. 0% and 4% in control group who was treated according to guidelines), suggesting that T2DM can be reversed if appropriate dietary and lifestyle interventions are applied shortly after diagnosis [71]. In a 48-month analysis of the same study weight loss of ≥15 kg was maintained by 11% and T2DM remission by 36% of participants of the intervention group. Importantly, 70% of those who maintained weight loss of ≥15 kg managed to achieve a durable remission, emphasizing the strong association between the two [72]. Similarly, the DIADEM-I randomized control trial, was designed to investigate the impact of intensive lifestyle intervention (total diet replacement and physical activity) on weight loss and T2DM remission but compared to DiRECT study it included younger patients (aged 18–50 years) with a shorter disease duration (≤3 years). Patients had a BMI of ≥27 kg/m^2^. After 12 months, the mean weight loss in the intervention group was 11.98 kg and remission of T2DM was achieved in 61% of the participants (compared to 3.98 kg and 12% in the control group who was treated with usual diabetes care) [73]. Βoth studies included patients not receiving insulin treatment at baseline, therefore without profound β-cell dysfunction, highlighting the importance of early interventions targeting obesity-driven insulin resistance.

In addition, whether restoration of β-cell function is possible if risk factors are controlled was investigated by several studies that included T2DM patients of all age ranges. Further metabolic investigations, including examinations for β-cell function, were performed on a subgroup of participants of the DiRECT study. On this study, Taylor et al. described a recovery of first-phase insulin secretion among the responder’s group, that was also sustained during the maintenance period, compared to no-change in the non-responders group. Responders vs. non-responders were defined by a shorter disease duration, highlighting that T2DM could be considered a reversible condition, if appropriate weight management interventions are applied early in the course of disease. Importantly, in the same study, ectopic adipose tissue content in liver and pancreas was decreased in both groups after weight loss [74]. However, further evidence is required regarding the extent of β-cell function reversibility, specifically in early-onset T2DM populations.

Importantly, in a considerable number of cases of adolescents with T2DM, adequate glycemic control cannot be achieved or maintained on a long-term basis if lifestyle interventions are applied alone [2, 75, 76]. Indeed, according to a study by Herbst et al. comprising 578 patients (mean age 15.5 ± 2.1 years), while regular physical activity is associated with lower HbA1c, higher high-density lipoprotein (HDL) and lower standardized BMI, it does not appear to de-escalate the required treatment regime [77].

Despite the rising incidence and faster progression of T2DM in younger ages, there are currently limited studies regarding oral hypoglycemic drugs in children and young adults. A Scandinavian population-based study from 2010 to 2019 aimed to examine the use of non-insulin antidiabetic drugs in patients <24 years and found that metformin was by far the most commonly prescribed medication in this age group, followed by glucagon-like peptide 1 (GLP-1) analogs, the use of which had an eightfold increase during the period that this study was conducted [78].

Pharmacotherapy options for patients <18 years are limited due to little existing evidence in regard to their safety and efficacy. Since 2000, metformin has been the only approved oral medication available for this age group. Empagliflozin, a sodium-glucose co-transporter-2 inhibitor that has revolutionized adult T2DM care in the past few years, was very recently approved by the US Food and Drug Administration for use in pediatric populations 10 years and older with T2DM [79]. Injectable GLP-1 agonists, liraglutide (daily) and exenatide extended release (once-weekly) have also been approved for T2DM treatment in the youth since 2019 and 2021 respectively [80, 81]. The rapid deterioration of β-cell function observed in early-onset phenotype usually leads to necessitation of insulin therapy at earlier stages in those patients compared to the later-onset T2DM (in >50% of patients by 2–5 years after diagnosis) [2, 60].

Results from the TODAY study, a multicentered randomized clinical trial combining lifestyle and drug treatment choices in 699 obese young patients (aged 10–17 years) with T2DM reported that metformin monotherapy resulted in durable glycemic control (HbA1c < 8% without requirement of insulin therapy) in only 50% of the participants, metformin plus rosiglitazone had superior results to metformin monotherapy and metformin combined to lifestyle intervention program had no difference to metformin alone. Further analysis of this study reported that treatment response differs in relation to ethnic origin and sex, with non-Hispanic Blacks having the poorest response and females having the best response to the combination treatment (metformin plus rosiglitazone) [82].

Moreover, with the rise in the prevalence of T2DM in young populations, including females of childbearing age, a rise in pregnancies complicated by type 2 diabetes is also anticipated. For instance, despite strong birth control recommendations on participants, 10.2% of female patients in the TODAY study experienced a pregnancy, with a considerable percentage resulting in unfavorable outcomes, such as pregnancy endings and major congenital abnormalities [83].

Another randomized clinical trial, The Restoring Insulin Secretion (RISE) Pediatric Medication Study, including 91 overweight or obese patients (aged 10–19 years) with IGT or newly diagnosed T2DM (<6 months), compared two different treatment approaches, 3 months of insulin glargine followed by 9 months of metformin or 12 months metformin alone, in order to evaluate the β-cell function during and after treatment. Neither of the two treatment alternatives succeeded in preventing β-cell function deterioration, emphasizing the need for new approaches aiming to maintain β-cell function [84].

Metabolic bariatric surgery is nowadays a common and effective treatment option for obese adults with T2DM. As for its role in management of youth obese populations with early-onset T2DM, there are currently limited, yet encouraging data available in regard to maintenance of glycemic control compared to non-surgical treatment [2, 85, 86]. Therefore, additional research in this field, including safety and long-term outcomes in this high-risk population is of utmost importance.

## Societal effects and challenges

Early-onset T2DM poses a number of challenges to public health care, distinctly different than if it presented later in life. Providing health care to those with an earlier diagnosis of T2DM can be challenging in terms of engaging with full-time working or studying populations, including women of reproductive age, in order to achieve appropriate glycemic targets. Additionally, Agarwal et al. in the context of the SEARCH for Diabetes Youth Study demonstrated that transferring from pediatric to adult care (or even no care, in 15%) resulted in worsening glycemic control due to loss of follow-up [87].

Moreover, a cross-sectional survey conducted in China showed that young adults with T2DM suffer much higher diabetes distress levels (90.82%) than elderly patients, which is amplified by social and economic-related responsibilities [88]. Recent data from the TODAY2 cohort study showed that low medication adherence is common in youth-onset T2DM patients (65.4%) and associated with certain need insecurities (e.g., housing) and interfering beliefs, such as that medications might be overused or harmful [89]. Recently, data from a study conducted by the Canadian Diabetes Association revealed that Covid-19 pandemic had great impact on the lives of adolescents and young adults living with T2DM, with only 70% of them having the availability of telephone or virtual clinic appointments with their healthcare provider, and most of them reporting worsening of their eating and exercising habits [90].

## Conclusions

DM is one of the major global public health challenges of the 21st century with a continuously rising prevalence and incidence. This review aimed to provide an overview of the up-to date knowledge with regard to early-onset T2DM.

Numerous modifiable and non-modifiable risk factors are reported to induce the early-phenotype disease presentation, among which, the escalating rates of overweight and obesity in pediatric and adolescent populations appear to be the most prominent. Furthermore, ectopic adiposity, especially in liver, is substantially higher in younger than older patients with T2DM and appears to be a key player of insulin resistance in this population. However, the striking difference with later-onset T2DM, is that the ability of pancreatic β-cells to compensate for the increased insulin resistance is early lost, highlighting the impairment of insulin secretion as the principal pathophysiologic mechanism. The exact pathophysiology pathways underpinning rapid β-cell decline are not clear; future research focusing on genetic, epigenetic, hormonal, and environmental parameters could shed more light on this phenomenon.

Apart from progressing faster, this clinical entity, compared to later-onset T2DM and T1DM, results in higher and earlier development of complications, especially microvascular, as well as increased relative morbidity and mortality. Importantly, these outcomes still remain not fully explained in terms of pathophysiology. Possibly, the increased prevalence of complications could be attributed to a more aggressive disease phenotype, given that it is also observed in studies controlled for diabetes duration and glycemic control. Nevertheless, long-term effects have only recently started to become apparent, and further research is required in order to specify the associated risk factors and develop appropriate prevention, screening, and management strategies.

For the time being, the therapeutic management of early-onset T2DM follows the same principles as in later-onset T2DM. Nevertheless, if a different pathogenesis is suspected, a different therapeutic management would seem plausible. Large, prospective, interventional controlled studies are required in order to develop treatment guidelines tailored to early-onset T2DM.

Last but not least, the challenges associated with social determinants in young populations have substantial impact on diabetes distress, low adherence to therapy and follow-up, prompting us to encompass structured psychosocial support and education in management of young patients with T2DM, following the paradigm of relevant strategies for T1DM patients.

In conclusion, early-onset T2DM is an emerging public health problem, with aggressive phenotype and great overall impact in life of the affected individuals. In order to optimize prevention and treatment strategies, it might be essential to reconceptualize it as a different form of DM; to this direction though, more pathogenetic and clinical aspects need to be elucidated.
